# Chromosome-scale genome of the human blood fluke *Schistosoma mekongi* and its implications for public health

**DOI:** 10.1186/s40249-023-01160-6

**Published:** 2023-11-28

**Authors:** Minyu Zhou, Lian Xu, Dahua Xu, Wen Chen, Jehangir Khan, Yue Hu, Hui Huang, Hang Wei, Yiqing Zhang, Phiraphol Chusongsang, Kanthi Tanasarnprasert, Xiang Hu, Yanin Limpanont, Zhiyue Lv

**Affiliations:** 1 Key Laboratory of Tropical Disease Control, Ministry of Education, Sun Yat-Sen University, Guangzhou, China; 2 Department of Pathogen Biology and Biosafety, Zhongshan School of Medicine, Sun Yat-Sen University, Guangzhou, China; 3 Key Laboratory of Neuroregeneration, Ministry of Education and Jiangsu Province, Co-Innovation Center of Neuroregeneration, NMPA Key Laboratory for Research and Evaluation of Tissue Engineering Technology Products, Nantong University, Nantong, China; 4 Key Laboratory of Tropical Translational Medicine of Ministry of Education, College of Biomedical Information and Engineering, Hainan Medical University, Haikou, China; 5 Key Laboratory of Vascular Biology and Translational Medicine, Medical School, Hunan University of Chinese Medicine, Changsha, China; 6 Hainan General Hospital, Hainan Affiliated Hospital of Hainan Medical University, Haikou, China; 7 Department of Social and Environmental Medicine, Faculty of Tropical Medicine, Mahidol University, Bangkok, Thailand; 8 State Key Laboratory of Developmental Biology of Freshwater Fish, College of Life Sciences, Hunan Normal University, Changsha, China

**Keywords:** *Schistosoma mekongi*, Chromosome-scale genome, Protease, RNA-seq

## Abstract

**Background:**

*Schistosoma mekongi* is a human blood fluke causing schistosomiasis that threatens approximately 1.5 million humans in the world. Nonetheless, the limited available *S. mekongi* genomic resources have hindered understanding of its biology and parasite-host interactions for disease management and pathogen control. The aim of our study was to integrate multiple technologies to construct a high-quality chromosome-level assembly of the *S. mekongi* genome.

**Methods:**

The reference genome for *S. mekongi* was generated through integrating Illumina, PacBio sequencing, 10 × Genomics linked-read sequencing, and high-throughput chromosome conformation capture (Hi-C) methods. In this study, we conducted de novo assembly, alignment, and gene prediction to assemble and annotate the genome. Comparative genomics allowed us to compare genomes across different species, shedding light on conserved regions and evolutionary relationships. Additionally, our transcriptomic analysis focused on genes associated with parasite-snail interactions in *S. mekongi* infection. We employed gene ontology (GO) enrichment analysis for functional annotation of these genes.

**Results:**

In the present study, the *S. mekongi* genome was both assembled into 8 pseudochromosomes with a length of 404 Mb, with contig N50 and scaffold N50 lengths of 1168 kb and 46,759 kb, respectively. We detected that 43% of the genome consists of repeat sequences and predicted 9103 protein-coding genes. We also focused on proteases, particularly leishmanolysin-like metalloproteases (M8), which are crucial in the invasion of hosts by 12 flatworm species. Through phylogenetic analysis, it was discovered that the M8 gene exhibits lineage-specific amplification among the genus *Schistosoma*. Lineage-specific expansion of M8 was observed in blood flukes. Additionally, the results of the RNA-seq revealed that a mass of genes related to metabolic and biosynthetic processes were up-regulated, which might be beneficial for cercaria production.

**Conclusions:**

This study delivers a high-quality, chromosome-scale reference genome of *S. mekongi*, enhancing our understanding of the divergence and evolution of *Schistosoma*. The molecular research conducted here also plays a pivotal role in drug discovery and vaccine development. Furthermore, our work greatly advances the understanding of host-parasite interactions, providing crucial insights for schistosomiasis intervention strategies.

**Graphical Abstract:**

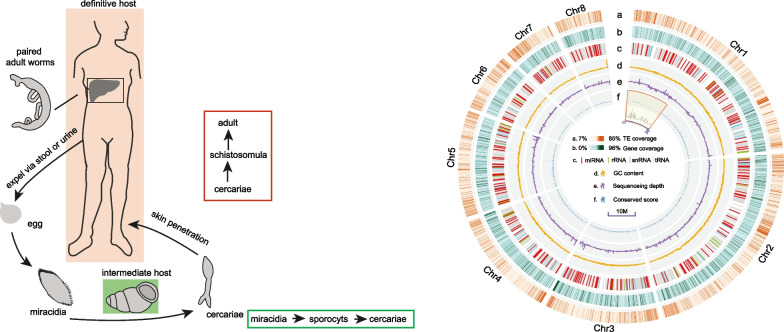

**Supplementary Information:**

The online version contains supplementary material available at 10.1186/s40249-023-01160-6.

## Background

Schistosomiasis (also known as bilharzia) is a tropical human disease caused by nematodes of the genus *Schistosoma* [1], which is considered the second most impacting human parasitic disease [2]. Annually, a global risk of 732 million infections and 280,000 fatalities has been estimated [3]. Mammalian hosts, including humans, are infected with schistosomes upon direct contact with cercariae-contaminated fresh water, enabling cercariae to get under the skin [3, 4]. Six major *Schistosoma* species have been identified so far to infect humans, including five well-known species (*Schistosoma japonicum*, *S. mansoni*, *S*. *guineensis*, *S. haematobium*, and *S. malayensis*) and two geographically localized species (*S. intercalatum* and *S. mekongi*) [5]. *S. mekongi* is mainly distributed in Cambodia, Myanmar, Thailand [6], Laos, and other regions near the Mekong River and its tributaries (Additional file 1: Fig. S1), where it poses a threat to about 1.5 million people [7]. A wide spread of *S. mekongi* associated with increased risk to the public's health has been recently predicted [8]. Individuals with *S. mekongi* infection (Mekong schistosomiasis) [9] usually exhibit a chronic inflammatory response, causing intestinal, hepatic, liver fibrosis [10], and splenic diseases [11], or even cancer [12]. Villagers are the main victims of *S. mekongi* in areas where it is endemic, but sporadic cases of infection among travelers and immigrants have also been reported, rendering this disease a significant travel medicine concern in Southeast Asia [9].

**Fig.1:**
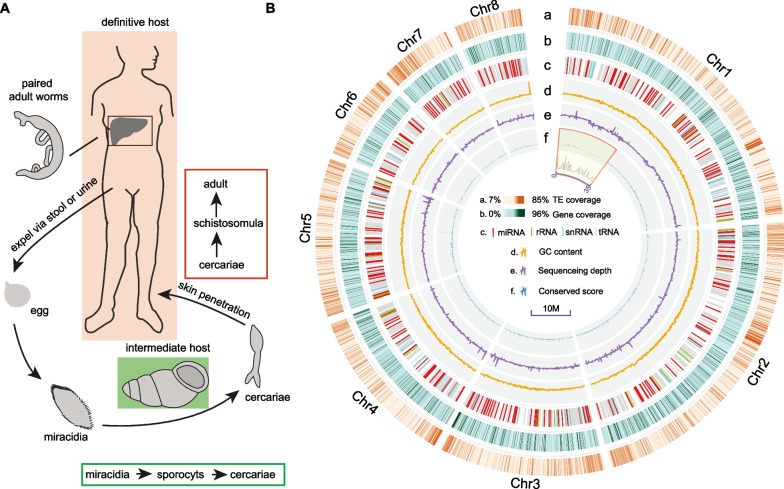
Life cycle and genomic characteristics of *S. mekongi*. **A** The life cycle of *S. mekongi* is similar to other blood flukes. Firstly, eggs are expelled from the definitive hosts (humans, dogs, pigs, cattle and rodents, etc.), then hatch and release miracidia under favorable conditions. Secondly, miracidia penetrate snail host tissue, develop into sporocysts, and are released as cercariae. Finally, cercariae encounter a definitive host, enter via skin penetration and develop into schistosomulae and subsequently adults; **B** genomic features of *S. mekongi*. Layers from outer to inner represent transposable elements (TEs) coverage (a), gene coverage (b), noncoding RNA (c), guanine-cytosine (GC) content (d), sequence depth (e) and conservation score (f). Blocks that are conserved among four human blood flukes [using chromosome 1 (Chr1) of *S. mekongi* as a reference] but conserved in three early-diverging species (*Trichobilharzia regenti*, *S. japonicum* and *S. mekongi*) are shaded in yellow in the inner circle

*S. mekongi* has a complicated lifecycle (Fig. 1A) similar to that of other blood flukes, requiring an intermediate snail host and a definitive host (such as humans). Specifically, cercariae are released from the snail host and then penetrate the skin of the mammalian host, where they develop into adult worms residing in the mesenteric veins. Male and female adult worms pair and produce eggs, which embolize host tissues such as the liver and intestines, resulting in granuloma and fibrosis. The eggs hatch after reaching freshwater and generate numerous miracidia via asexual replication. The miracidia then invade snail intermediate hosts, particularly *Neotricula aperta*, where they further develop into cercariae in preparation for a new round of infection [13, 14]. Due to the unavailability of effective vaccines against *S. mekongi*, praziquantel (PZQ) is currently the most effective drug for schistosomiasis management, although mass drug administration appears to have decreased its efficacy [15]. Therefore, it is indispensable to find new effective targets (through genomic and proteomic analysis) for accelerating drug discovery and vaccine development [16]. Recent progress in sequencing technology has disclosed high-quality genome resources for human blood flukes including *S. mansoni* [17], *S. haematobium* [18–20], and *S. japonicum* [14, 21, 22]. Although the development and morphological features of *S. mekongi* are similar to those of other blood flukes [23, 24], whole-genome information for this species is still lacking, preventing the exploration of the molecular mechanisms underlying the adaptation, evolution, and genetic manipulation of schistosome species.

In this study, we present a chromosome-level assembly of the *S. mekongi* genome by integrating Illumina, PacBio sequencing, 10 × Genomics linked-read sequencing, and high-throughput chromosome conformation capture (Hi-C) technologies. The quality of the obtained genome assembly was assessed by Benchmarking Universal Single-Copy Orthologs (BUSCO) analysis and compared with the other available schistosome genomes. The assembly had a total size of 404 Mb, with contig and scaffold N50 values of 1168 kb and 46,759 kb, respectively. 93.74% of the sequences could be anchored to eight pseudomolecules. In addition, we employed comparative analysis against other flatworms to explore potential parasitism-related molecules in blood flukes and conducted an investigation of transcriptional changes in snails with miracidia infection. Collectively, this *S. mekongi* genome dataset could serve as a valuable resource for subsequent transcriptome analysis, comparative genomics studies across different schistosome species, and the development of schistosomiasis treatments. The geographic distribution of *S. mekongi* closely overlaps that of the intermediate host, consequently, the distribution of *N. aperta* is responsible for the prevalence of Mekong schistosomiasis [85–87]. In the context of “one health,” studying the transcriptome of snails will be beneficial for controlling the transmission of Mekong schistosomiasis. RNA-seq was conducted to explore the host-parasite interactions in the present study.

## Material and methods

### Ethics statement

All protocols involving animals were conducted following the guidelines of the Association for Assessment and Accreditation of Laboratory Animal Care International. The experimental procedures were in accordance with the institutional ethical guidelines approved by the ethics committee at the Zhongshan School of Medicine, Sun Yat-Sen University. All animal use for *S. mekongi* sample preparation in this study was approved by the Faculty of Tropical Medicine-Animal Care and Use Committee, Mahidol University (approval number FTM-ACUC 002/2018E).

### Sample preparation

*S. mekongi* was maintained in the freshwater snail *Neotricula aperta* as an intermediate host and in ICR mice as the final host at the Applied Malacology Laboratory, Faculty of Tropical Medicine, Mahidol University.

To obtain *S. mekongi* egg samples, the livers and fecal-free intestines of infected mice collected after eight weeks of infection were homogenized using a Waring blender and washed through a column of sieves (420-, 177-, 105-, and 44-µm mesh openings, in order) with 0.85% saline. The eggs were washed through the bottom sieve, rinsed 2–3 times, and then stored at – 20 ℃ for later use.

To prepare the cercarial stage of *S. mekongi*, *N. aperta* snails were individually exposed to ten miracidia hatched from the eggs. Exposure was carried out by placing a snail in a 94-well plate containing miracidia under light for 2–3 h. The snails were maintained in Petri dishes for eight weeks and then examined for the presence of cercaria using the light shedding method [25]. The cercariae were then collected and stored at – 20 ℃.

To obtain adult-stage samples, the ICR mice were infected with cercariae (20–30 cercariae/mouse) by abdominal skin exposure. After eight weeks of infection, the mice were euthanized via the inhalation of CO_2_. Then, the mice were dissected, and adult flukes were collected by perfusion using a 0.85% saline solution. The solution containing the adult worms was transferred to a sedimentation glass. Adult worms that settled to the bottom of the glass were collected and stored at – 80 ℃ for later use. All animal use in this study was approved by the Faculty of Tropical Medicine-Animal Care and Use Committee, Mahidol University (approval number FTM-ACUC 002/2018E).

### DNA extraction, library construction and sequencing

Illumina paired-end sequencing, PacBio sequencing, 10 × Genomics linked-read sequencing and Hi-C technology were combined to yield a high-confident genomic library. For Illumina paired-end sequencing, libraries were generated using the NEB Next^®^ Ultra DNA Library Prep Kit for Illumina^®^ (NEB, Ipswich, MA, USA) following the manufacturer’s recommendations. Briefly, DNA was purified using the AMPure XP system (Beckman Coulter, Beverly, USA). After the adenylation of the 3′ ends of DNA fragments, a NEBNext Adaptor with a hairpin loop structure was ligated to prepare for hybridization. Then, electrophoresis was used to select DNA fragments with specified lengths. Three microliters of USER Enzyme (NEB) were incubated with size-selected, adaptor-ligated DNA at 37 ℃ for 15 min and then at 95 ℃ for 5 min. Thereafter, PCR was performed with Phusion High Fidelity DNA polymerase, universal PCR primers and an Index (X) Primer. Finally, the PCR products were purified using AMPure XP system (Beckman Coulter Inc., Brea, CA, USA), and library quality was assessed on an Agilent Bioanalyzer 2100 system. Sequencing was performed on the HiSeq X-ten platform (Illumina, CA, USA).

For PacBio sequencing, libraries were constructed following the standard protocols of the Pacific Biosciences company (California, USA). Briefly, high-molecular-weight genomic DNA was sheared to a size of ~ 20 kb, followed by damage repair and end repair, blunt-end adaptor ligation, and size selection. Finally, the libraries were sequenced on the PacBio Sequel platform at the Novogene Co., Ltd (Beijing, China).

For 10 × Genomics linked-read sequencing, the Read 1 sequence and the 10X™ barcode were added to the molecules during gel bead-in-emulsion (GEM) incubation. P5 and P7 primers, Read 2, and the sample index were added during library construction via end repair, A-tailing, adaptor ligation, and amplification. The final libraries contained the P5 and P7 primers were used in Illumina® bridge amplification, and sequenced on the HiSeq X-ten platform.

For Hi-C sequencing, chromatin was digested with 400 U of the *Hin*dIII restriction enzyme (NEB) at 37 ℃ for 16 h. DNA ends were labeled with biotin and incubated at 37 ℃ for 45 min, and the enzyme was inactivated with a 20% SDS solution. DNA ligation was performed by adding T4 DNA ligase (NEB) and incubation at 16 ℃ for 4–6 h. After ligation, proteinase K was added to reverse cross-linking after ligation, followed by incubation at 65 ℃ overnight. DNA fragments were purified and dissolved in 86 μl of ultrapure water, and unligated ends were removed. The purified DNA was fragmented to a size of 300–500 bp, and DNA ends were then repaired. DNA fragments labeled by biotin were finally separated with Dynabeads^®^ M-280 Streptavidin (Life Technologies, Waltham, MA, USA). The Hi-C libraries were subjected to quality control and sequenced on a HiSeq X-Ten sequencer.

### Genome assembly and quality control

Wtdbg (v1.2.8) [26] was first used to assemble the PacBio reads. The assembled contigs were further polished with PacBio reads using Quiver (implemented in SMRTlink v5.0.1) (https://www.pacb.com/support/software-downloads) and corrected with Illumina reads using Pilon (v1.22) [27]. Next, 10 × Genomics long reads were employed to build scaffolds using fragScaff (v140324) [28]. Finally, Hi-C reads were employed to anchor the scaffolds to chromosomes using Lachesis (v201701) [29]. BLAST + (v2.6.0) [30] was used to map the assembly to the Nucleotide Sequence Database (ftp://ftp.ncbi.nih.gov/blast/db, accessed in August 2020). BWA (0.7.15) was used to map Illumina genomic reads against references with the default parameters [31]. BlobTools (v1.1.1) was used to detect contamination [32]. Genome assembly quality and annotation completeness were assessed using BUSCO (version 5.2.1) [33] with the data set eurotiomycetes_odb10.

### Mitochondrial genome assembly of *S. mekongi*

To assemble the mitochondrial genome, the corresponding PacBio reads were extracted by using BLASTN [30] and compared with the reference sequence of the *S. mekongi* mitochondrion (NCBI Accession No. NC_002529.1). The filtered reads (alignment length > 5 kb and identity > 75) were then assembled using wtdbg2 (v1.2.8) [26] and qualified. After assembly, the Illumina reads were used for error polishing with Pilon (v1.22) [27]. We manually trimmed the overlapping part of the mitochondrial contig was filtered manually and circularized it. Finally, the mitochondrial genome was annotated using GeSeq [34] and visualized using OGDRAW [35].

### Repeat analysis

Full-length long terminal repeats (LTRs) from five blood fluke genomes were predicted using LTR_STRUC [36], and a pair of target site duplications (TSDs) in an LTR was extracted, MUSCLE [37] was employed to align the pair of TSDs, and “Dismat” from the EMBOSS package [38] was used to estimate divergence distances. InterProscan [39] was employed to identify Pfams in LTRs. To reduce computation time and illustrate the evolution of homologous LTRs, we first used orthoMCL [40] to exclude singletons and low-copy-number sequences. Sequences with more than 20 copies in a cluster were extracted, and multiple-sequence alignment was performed with MUSCLE with the default parameters. Poorly aligned regions were removed using TrimAl [50] with the “-automated1” parameters. FastTree (v2.1.11), a rapid phylogenetic reconstruction tool [51], was used to reconstruct the phylogeny, among five blood flukes.

### Genomic and functional annotations

Repeat identification was performed by combining de novo and homolog-based strategies. TRF (v4.09) was used to detect simple tandem repeats [41]. Homolog-based prediction was performed by RepeatMasker (v4.07) (http://www.repeatmasker.org/) and Proteinmask package (v4.07) in RepeatMasker. For de novo prediction, we used RepeatModeler (v2.0.1) (http://www.repeatmasker.org/RepeatModeler/), Piler (v1.0) [42], and RepeatScout (v1.0.5) [43] to construct a de novo library and used RepeatMasker to annotate transposable elements (TEs).

To annotate protein-coding models in the assembly, we integrated evidence from homolog-based-, de novo*-* and RNA-seq-assembled transcripts. Briefly, the RNA-seq reads of the worms (including female adults, male adults, and eggs) were aligned to the genome with TopHat (v2.0.13) [44] and then assembled into transcripts using Cufflinks (v2.1.1) [45]. We also performed the de novo assembly of RNA-seq reads using Trinity (v2.1.1) [46] and conducted further assembled using PASA (v2.4.1) [47]. Augustus (v3.2.3) [48], Genscan (v1.0) [49], Geneid (v1.4) [50], GlimmerHMM (v3.0.4) [51] and SNAP (v2013.11.29) [52] were employed for de novo prediction. Proteins from six closely related species were mapped to the genome using BLASTP (v2.2.26) [30], and high-scoring blocks were joined for each gene pair using Solar (v0.9.6) [53]. Genewise implementation in Wise2 (v2.4.1) (http://www.sanger.ac.uk/Software/Wise2/) was used to realign each matched protein and genomic sequence to define gene structure. EVidenceModeler (EVM, v1.1.1) [54] was used to combine de novo prediction, homology-based and RNA-seq evidence to construct gene models. Finally, PASA was used to correct EVM-predicted gene models and provide supplemental information on splicing sites and untranslated region (UTRs). The final gene models were annotated by searching against databases Swiss-Prot (http://www.expasy.ch/sprot), Nr (http://www.ncbi.nlm.nih.gov), Pfam (http://pfam.xfam.org/), KEGG (https://www.genome.jp/kegg/kegg2.html) and InterPro (https://www.ebi.ac.uk/interpro/). Putative signal peptide and transmembrane domains were predicted using the programs SignalP (v4.1) [55] and TMHMM (v2.0c) [56]. Classical ES proteins were inferred based on the presence of signal peptide domains and the absence of transmembrane domains. The cellular location of each putative ES protein was predicted using MultiLoc2 (http://abi.inf.unituebingen.de/Services/MultiLoc2). Putative peptidases or inhibitors were detected by searching against the MEROPS [57] peptidase unit database using BLASTP.

Non-coding RNAs (tRNA, rRNA, miRNA, and snRNA) were searched against public databases, among which tRNA was detected using tRNAscan-SE (v2.0.6) [58], rRNA was determined using BLAST, and miRNA and snRNA were identified using Infernal [59].

### Multiple genome alignment

Four repeat-soft-masked whole *Schistosoma* genomes were first collected. The genome of *S. mekongi* was considered a reference sequence when applying LASTZ (v1.04.00) [60] for alignment with each of the other three genomes separately. Then, the multiple genome alignment among human blood flukes was analyzed using MULTIZ (v11.2) [61] based on the LASTZ results. The aligned sequences were finally analyzed using PhastCons (v0.9.9.10b) [62] to detect conserved regions across the genomes using *S. mekongi* as a reference. The detailed methods were consist as described previously (https://github.com/gigascience/paper-zhang2014, https://github.com/JinfengChen/Genomes). The genes located in conserved elements were determined by using BEDTools [63].

### Phylogenetic analysis of cercarial elastases (CEs) and M8

We downloaded experimental evidence of CEs in flatworms and performed BLAST analysis based on available genomes we obtained. Candidate high-scoring segments were concatenated using Solar, and gene structures were defined using Genewise [64]. M8 candidates from 12 species were annotated in searching against sequences deposited in MEROPS.

To reconstruct the phylogeny of CEs and M8, proteins were subjected to multiple alignment using MUSCLE, and poor alignments were trimmed using TrimAl. IQ-TREE (v1.6.12) [65] was used to determine the best model and reconstruct the phylogeny.

### De novo assembly and functional annotation of snail transcriptome

The clean reads of one naive snail and *S. mekongi*-infected snail were obtained from Illumina. The de novo assembly of clean reads was performed by Trinity (v2.8.5) [46]. We removed redundancy by retaining the longest contig if several contigs were annotated to the same gene. Hisat2 (v7.5.0) [66] was used to map clean reads onto the genome of *S. mekongi*, and mixed reads in the snail transcriptome belonging to *Schistosoma* were excluded. TransDecoder (v5.5.0) [10] was used to predict coding regions and proteins based on the genomes of *Biomphalaria glabrata* (https://vectorbase.org) and *Pomacea canaliculata* (http://gigadb.org/dataset/view/id/100485/). The remaining contigs were defined as unigenes, and their expression levels were calculated using RSEM [67] according to the corresponding fragments per kilobases per million reads (FPKM) values. To identify differentially expressed genes, GFOLD (v1.1.4) [68] analysis was conducted following the threshold of GFOLD values > 2 were considered significant. Finally, the GO enrichment analysis of was performed using Trinotate (v3.2.0) (http://trinotate.github.io/) (Broad Institute, Cambridge, MA, USA) to identify the functional annotation of genes (http://geneontology.org/).

### Data availability

All raw sequencing data generated here have been deposited in the public database NCBI-Sequence Read Archive (SRA), and annotated genome assembly results have been uploaded to GenBank under the bioproject number PRJNA803609. RNA-seq for the *T. regenti* (PRJNA292737), *O. hupensis* (PRJNA551328) and *S. mekongi* (SRP136896) [9] were retrieved from National Center for Biotechnology Information (NCBI).

## Results

### Assembly and annotation of the *S. mekongi* genome

To obtain a high-quality reference assembly of the human blood fluke *S. mekongi*, we sequenced its genome by integrating Illumina (80-fold coverage), PacBio (102-fold coverage) and 10 × Genomics (296-fold coverage) technologies (Additional file 1: Table S1). The estimated genome size and heterozygosity rate of *S. mekongi* were 419.07 Mb and 0.27%, respectively, based on 17-kmer estimation using Illumina reads (Additional file 1: Fig. S2 and Table S2). PacBio reads were assembled into initial contigs and then polished with PacBio and Illumina reads. The 10 × Genomics linked reads were further scaffolded into contigs. To obtain the chromosome-level assembly, we anchored scaffolds onto chromosomes with Hi-C. The total length of the final assembly was 404 Mb and was anchored to eight pseudomolecules (93% of the estimated genome size, Additional file 1: Fig. S3), with contig N50 and scaffold N50 lengths of 1168 kb and 46,759 kb, respectively (Table 1). This assembly is among the best obtained for the genus to date, second only to that of the model species *S. mansoni*. No obvious contamination was detected in the assembly (Additional file 1: Fig. S4) and approximately 97.53% of the Illumina reads could be mapped to the assembly. The GC content (33.73%) of *S. mekongi* was similar to those of other schistosome species, including *S. mansoni* (https://parasite.wormbase.org/Schistosoma_mansoni_prjea36577/Info/Index), *S. japonicum* (https://ngdc.cncb.ac.cn/bioproject/browse/PRJCA010213) and *S. haematobium* (Genbank accession number GCA_000699445.3) (Fig. 1B, Table 1 and Additional file 1: Fig. S5). The BUSCO pipeline is widely used for the assessment of assembly completeness by examining the coverage of highly conserved genes. The BUSCO results of assessment in five schistosomes showed that the completeness of our assembly harboring 86.7% aligned core eukaryote genes (Additional file 1: Table S3 and Fig. S6). The relatively low completeness (85.8–89.8%) of the schistosome genomes obtained to date may be due to divergence from the sequences of species deposited in databases (BUSCO). We also assembled the mitochondrial genome of *S. mekongi* using PacBio long reads. The resulting circular mitochondrial (mt) contig had a length of 14,360 bp (Additional file 1: Fig. S7) and was approximately identical to the mt sequence deposited in the GenBank database (accession NC_002529.1).

**Table 1:** Characteristics of four human blood flukes (*Schistosome mekongi*, *S. mansoni*, *S. japonicum*, and *S. haematobium*) and their genome information

Genomic features	*S. mekongi*	*S. mansoni*	*S. japonicum*	*S. haematobium*
BioProject	This study	PRJEA36577	PRJCA010213	PRJNA78265
Genome size (Mb)	404	391	406	400
Number of scaffolds	1285	10	100	163
Scaffold N50 (bp)	46,759,691	46,471,573	49,539,924	48,328,128
Number of contigs	1921	366	655	208
Contig N50 (bp)	1,168,079	4,678,053	2,378,822	20,231,032
GC content (%)	34.81	35.47	33.92	35.16
Number of protein coding genes	9103	9920	9760	9431

**Fig. 2:**
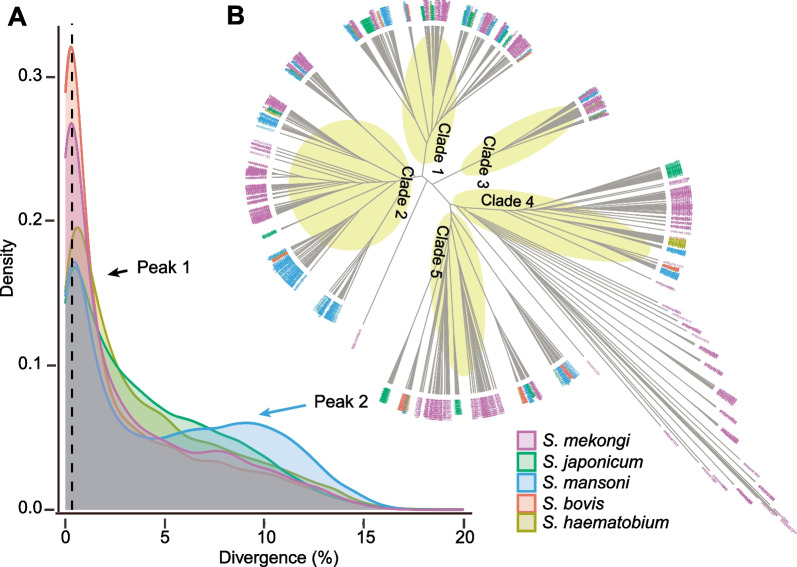
Long terminal repeat (LTR) divergence and evolution in the genomes of five schistosomes. **A** Divergence of full-length LTRs in five schistosomes; **B** phylogenetic relationships of reverse transcriptase (RT) domains in five schistosomes. Five major clades are shaded in yellow

A high proportion of repeats (43% of the genome) was found in the *S. mekongi* genome, 22% of which were long interspersed nuclear elements (LINEs), while approximately 4.66% were LTRs. For transposable elements (TEs), the RTE-BovB (14.37%) and Gypsy (4.26%) subtypes were the most abundant in the LINE and LTR clades, respectively (Additional file 1: Tables S4 and S5). Our repeat estimate was close to the proportion reported previously in *S. mansoni* (40%) [69]. TSDs are present in LTRs and enable the identification of young or undiverged complete LTRs; hence, the divergence of TSDs can aid in illustrating their evolution. To trace the evolution of LTRs in five blood fluke species, we first identified the complete LTRs in their genomes via the same pipeline using the LTR_STRUCT program. We identified 4452–9007 LTRs in the genomes, including 7338 copies (9.74% of the genome) in *S. mekongi*, which was greater than the number identified in *S. japonicum* (4452 copies, 6.95% of the genome). The distance of the TSDs calculated with “Dismat” implemented in the EMBOSS package showed that the five blood flukes presented a recent burst of LTR expansion, with a peak at a distance of less than 1% (peak 1 in Fig. 2A); this was particularly evident in *S. mekongi* and *S. bovis*, but an additional relatively old peak (approximately 10%, peak 2 in Fig. 2A) was found in *S. mansoni*.

To understand the evolution of LTRs, we extracted reverse transposase (RT) domains and then excluded divergent copies using the orthologous clustering method (clusters with at least 20 copies in five blood flukes were retained). The maximum likelihood estimated phylogeny showed that five major clades containing LTRs were derived from these five blood flukes, some of which represented species-specific expansions (Fig. 2B). These results suggested that these copies may have been inserted into the ancestral genome and then undergone expansion and divergence after speciation. Regarding noncoding RNAs, we predicted 255 miRNAs, 427 tRNAs, 806 rRNAs and 639 snRNAs (Fig. 1B and Additional file 1: Table S6).

A total of 9103 protein-coding genes were predicted by combining homolog-based RNA-seq evidence and de novo methods. A total of 8864 (97.4%) genes were supported by RNA sequencing [those with expression levels represented by FPKM values ≥ 0.1 were counted]. The distribution of various gene structures [gene length, coding sequence (CDS) length, intron length and exon length] was similar among the five blood flukes (Additional file 1: Fig. S8). At least 8370 (99%) proteins matched entries in the Swiss-Prot, Nr, Pfam (6414 genes), InterPro, GO (5499 genes), and KEGG databases (6906 genes). A total of 1979 proteins contained a transmembrane domain, and 565 possessed signal peptide sequences. A total of 316 secreted proteins were predicted based on the presence of a signal peptide and the absence of a transmembrane domain. (Additional file 1: Table S7). We also identified 355 peptides (including 89 cysteine peptidases, 93 metallopeptidases and 83 serine peptidases) and 108 peptidase inhibitors (Additional file 1: Table S8). Previously, some studies implicated these proteins in the life cycle of schistosomes as well as in host-parasite interactions [9, 70–72]. Our investigation offers diverse peptidases in *S. mekongi,* and thus, we suggest that components of these processes represent prospective new therapeutic targets for the treatment of schistosomiasis, as exploited by others [73].

### Genomic conservation estimation in four human blood flukes

The high-quality genomes of four human blood flukes (*S. mekongi*, *S. mansoni*, *S. haematobium* and *S. japonicum*) enabled us to investigate genome conservation. We employed whole-genome alignment combined with phylogenetic information (Multiz) to detect conserved genomic regions and then calculated conservation scores using PhastCons. Conserved regions comprising a total of 4,044,887 bp were identified in the four blood flukes, including 4046 genes (44.4%) in *S. mekongi*. GO enrichment results showed that these genes were involved in signal transduction and the regulation of cellular processes (Additional file 1: Table S9). Interestingly, we also found a 27.8 Mb region Chr 1 (or Chr ZW in *S. mansoni*), containing 650 genes, with extremely low conservative scores compared to the average value (0.4934, Fig. 1). This low-conservation region was conserved in *S. japonicum* and *S. mekongi* but was poorly conserved in the other two species. To investigate whether this region originated in the common ancestor of schistosomes, we performed the same analysis with the addition of a bird schistosome, *Trichobilharzia regenti* (Schistosomatidae), which is regarded as the closest relative to the schistosomes. The results showed relatively high conservation in these three species, representative of basal schistosomes (Fig. 1B). GO enrichment analysis showed that genes in this low-conservation region were associated with terms such as negative regulation of macromolecule metabolic process (Additional file 1: Table S9). Public RNA-seq data (four biological replicates) from cercariae (definitive host invasion) and schistosomulae (parasitic in definitive host) in *T. regenti* were employed to infer the potential functions of these genes at two important stages. A total of 447 out of 650 genes showed one-to-one orthologs in *S. mekongi* and *T. regenti*, and 285 out of 447 genes showed significant differential expression between cercaria and schistosomulae (65 up-regulated genes in cercaria and 220 up-regulated genes in schistosomulae, Fig. 5B). We further examined these genes by detecting one-to-one orthologs in *S. mansoni*. A total of 513 genes showed one-to-one interaction between *S. mansoni* and *S. mekongi,* and most of the identified genes were located on Chr ZW (Chr 1 in *S. mekongi*).

### Genome evolution

**Fig. 3:**
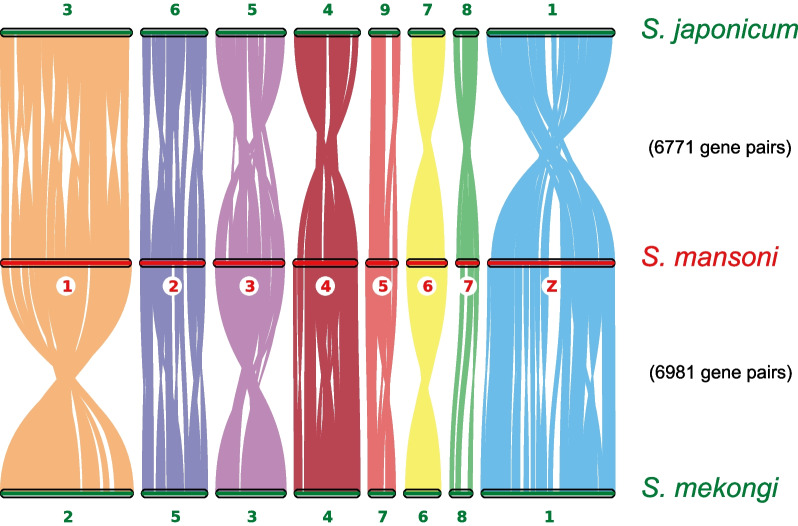
Genomic synteny among three human schistosome species inferred from orthologous gene pairs. Orthologous anchors were identified and visualized with the MSCANX program. A total of 6771 orthologous pairs were identified between *S. japonicum* and *S. mansoni,* and 6981 orthologous pairs were identified between *S. mansoni* and *S. mekongi*. *S. mansoni* chromosomes were used as a baseline, and scaffolds from *S. japonicum* were ordered based on *S. mansoni.* Different colors indicate different chromosomes of *S. mansoni*

We next investigated genome synteny in three human flukes (*S. mansoni*, *S. mekongi*, and *S. japonicum*) by considering assembly completeness and their relationships during evolution. We used the genome of *S. mansoni* schistosomulae (an extensively studied model species of schistosome) as a reference and compared it to the genomes of *S. mekongi* and *S. japonicum* using orthologs as anchors. The chromosomes or scaffolds of *S. mekongi* and *S. japonicum* were reordered based on the chromosomes of *S. mansoni* (Fig. 3). The results showed macro-synteny at the chromosome level, although local rearrangement and inversion existed within chromosomes (e.g., ZW and Chr5 in *S. mansoni*).

**Fig. 4:**
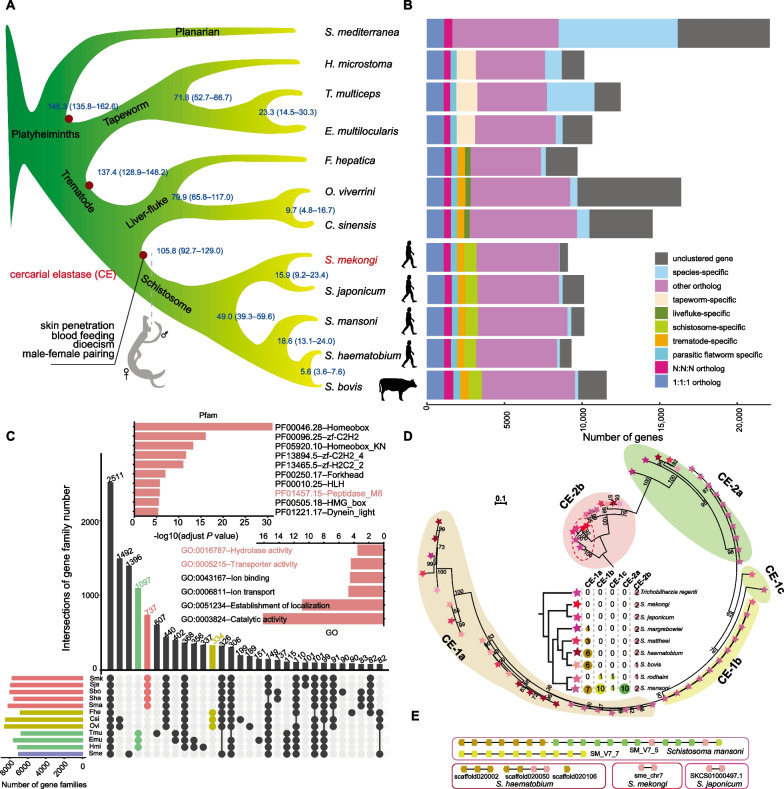
Phylogenetic relationships and genomic comparison of 12 species of flatworms. **A** Phylogenetic relationships of 12 species of flatworms. The red dots (for calibration) represent the divergence times retrieved from the study; **B** summary of gene family clustering inferred by using orthoMCL. 1:1:1 orthologs include the shared orthologs with the same number of copies in different species, N:N:N orthologs include the shared orthologs with different copy numbers in different species, patchy orthologs include the orthologs existing in at least one species, other orthologs include the unclassified orthologs, and unclustered genes include the genes that cannot be clustered into any cluster; **C** summary of shared gene family numbers in 12 species; **D** phylogenetic relationships of blood fluke-specific cercarial elastases (CEs) and their gene loci on scaffolds; **E** CE loci in four relatively good assemblies of schistosome species

We next performed a comparative analysis with other flatworms (free-living planaria, parasitic tapeworms, and liver flukes). Proteomes from 12 species (including free-living *Schmidtea mediterranea*, three parasitic tapeworms, three liver flukes, and five blood flukes) were clustered into 16,496 gene families. A phylogeny based on 1121 single-copy genes showed that *S. mekongi* was sister to *S. japonicum* (Fig. 4A). Divergence time estimation showed that the split of *S. japonicum* and *S. mekongi* occurred approximately 15.9 million years ago (MYA, Fig. 4A). Gene family clustering analysis showed that 737 gene families (778 genes in *S. mekongi*) were schistosome-specific (Fig. 4B and C). GO enrichment in *S. mekongi* showed that terms related to transporter activity and hydrolase activity [e.g., cercarial elastase (CE)] were distinctively altered (adjusted* P* < 0.05, Fig. 4C and Additional file 1: Table S10). CE is the major invasive larval protease in schistosomes and contributes to worms’ skin penetration by worms and exit from the intermediate snail host [14]. In addition, CEs have been reported to show unique expansion in *S. mansoni*. Recently released helminth genomes enable us to investigate the evolution of this protease in more detail, particularly in the bird blood fluke *T. regent* [74]. Hence, we added four other flukes (*T. regenti*, *S. margrebowiei*, *S. rodhaini*, and *S. mattheei*) to the analysis and performed genome-wide searches against the 16 species. Homologues of CEs were not detected in non-blood flukes, indicating that CEs are indeed blood fluke-specific and originated in the last common ancestor of Schistosomatoidea. Two copies of CEs were detected in three early-diverging blood fluke species (*T. regenti*, *S. mekongi*, and *S. japonicum*), which arose via tandem duplication (Fig. 4D). More than two copies of CEs were detected in other blood flukes, and substantial expansion was found in *S. mansoni* (~ 30 copies, Fig. 4D). Phylogenic analysis and classification based on previous classification in *S. mansoni* (CE-2a, CE-2b, CE-1a, CE-1b and CE-1c) showed the specific expansion of CE-1a in the other five schistosomes (Fig. 4D). CE-2a was uniquely present in *S. mansoni*, CE-1c and CE-1b were only present in the last common ancestor of *S. mansoni* and *S. rodhaini*, and CE-1b underwent a specific expansion in *S. mansoni* (Fig. 4D). Gene loci indicated that the expansion of CEs in schistosomes occurred mainly through tandem duplication (Fig. 4E).

### Lineage expansion of leishmanolysin-like metalloproteases (LeishLMPs) in blood flukes

**Fig. 5:**
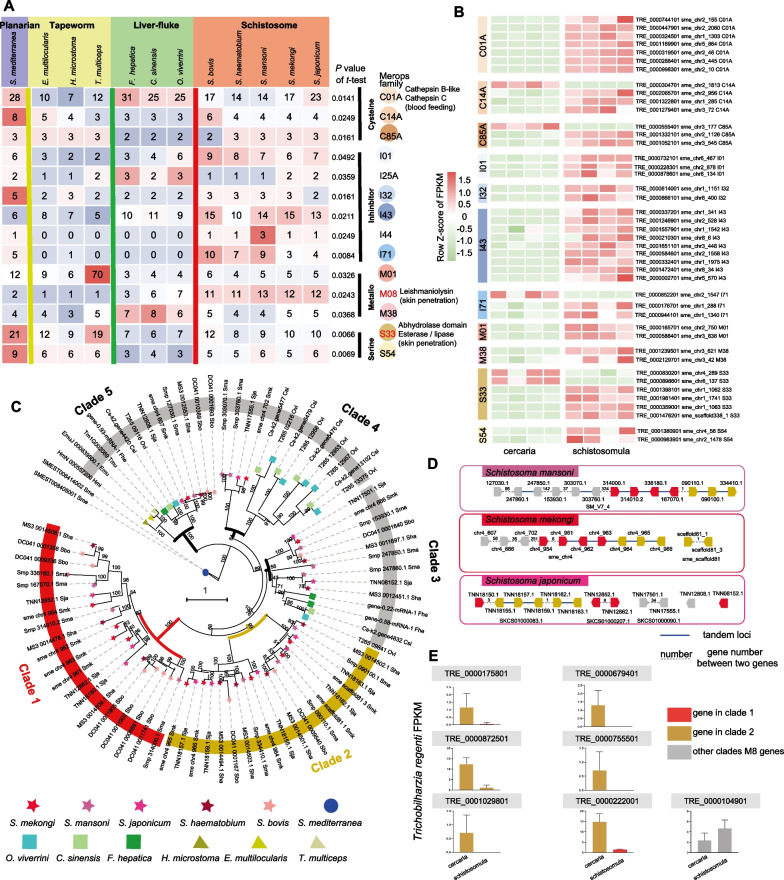
Proteases and phylogenetic analysis of leishmanolysin-like metalloproteases (M8) in 12 flatworms. **A** Different numbers of protease and inhibitor protein-coding genes between blood flukes and liver flukes; **B** gene expression profiles of proteases and protease inhibitors between cercariae and schistosomulae of *T. regenti* with one-to-one orthologs in *S. mekongi* based on reciprocal BLAST identification; **C** evolution of M8 in 12 flatworms. Two clusters of *Schistosoma* genus-specific expanded M8 sequences are highlighted in red and yellow; **D** M8 loci in three schistosome species; **E** expression values (Fragments Per Kilobase Million, FPKM) of M8 in cercariae and schistosomulae of an avian blood fluke (*T. regenti*)

Proteases and protease inhibitors play vital roles in host invasion, tissue migration, immune modulation and feeding [75]. We annotated and compared proteases and protease inhibitors in 12 flatworms using the same pipeline by searching against genes deposited in the MEROPS database (Additional file 1: Table S8). We focused on proteases and protease inhibitors showing different numbers between liver flukes and blood flukes to identify potential targets related to their different biological traits, such as tissue invasion and dwelling. In total, 14 families (8 belonging to proteases and 6 belonging to protease inhibitors) showing significant differences were detected (Student’s *t* test, *P* < 0.05, Fig. 5A). Compared with liver flukes, the number of genes encoding M8 (leishmanolysin-like metalloprotease), S33 (lysosomal acid lipase/cholesteryl ester hydrolase), I71 (*P* = 0.0084), and I01 (*P* = 0.0492) significantly increased in the blood flukes, whereas the number of genes encoding C01A (cathepsin) (*P* = 0.0141) and M38 (*P* = 0.0368) significantly decreased in the blood flukes. This disparity in differential gene/protein expression could be exploited for a promising anti-schistosome remedy, as used by others [76, 77].

Cathepsin L in *S. mansoni* (SmCL1, 2, and 3) is located in gastrodermal cells in the parasite cecum and functions by degrading host haemoglobin to meet its nutrient demands [78]. Cathepsin-B (subject to C01A) is involved in the uptake of erythrocytes by schistosomes [79] and participates in excystment and migration to hosts in the early stage of liver flukes [80]. The expression profiles of cercariae and schistosomulae in *T. regenti* (only 1:1 orthologs between *T. regenti* and *S. mekongi* were considered) showed that most proteases were significantly upregulated in schistosomulae dwelling in definitive hosts (Fig. 5B), including cathepsin B-like [81], cathepsin C, and cathepsin L proteases, suggesting that they might play key roles in blood feeding in schistosomes.

Our findings showed that genes encoding SmLeish were distinctively increased in cercaria and revealed that only 1–2 copies of M8 were present in planaria and tapeworm (Clade 5 in Fig. 5C), while multiple copies were displayed in flukes (3–7 copies in liver flukes and 11–13 copies in blood flukes), and complex evolutionary traces existed in flukes (two or three clades in liver flukes and five clades in blood flukes, Fig. 5C). Lineage-specific expansion of M8 (Clade 1 and Clade 2 in Fig. 5C) was observed in blood flukes. The analysis of M8 gene loci in blood flukes showed that lineage-specific M8 was probably generated via tandem duplication, as inferred by the examination of M8 loci at the chromosome scale in *S. mansoni* and *S. mekongi* (Fig. 5D). The M8 expression profiles of *T. regenti* cercariae and schistosomulae showed that the expression of linage-specific M8 copies was nearly restricted to cercariae, indicating that these metalloproteases might be related to the invasion of definitive hosts. Moreover, these genes were similar to SmLeish (belonging to Clade 3 shown in Fig. 5C), which exhibited high expression in sporocysts and is beneficial for larval survival in intermediate hosts. Both liver flukes and blood flukes require an intermediate snail host to develop into cercariae. The presence of M8 in clades 1–4 might be related to their survival in snails by helping to overcome snail immune cell attacks.

### Gene expression changes in snails respond to blood fluke infection

**Fig. 6:**
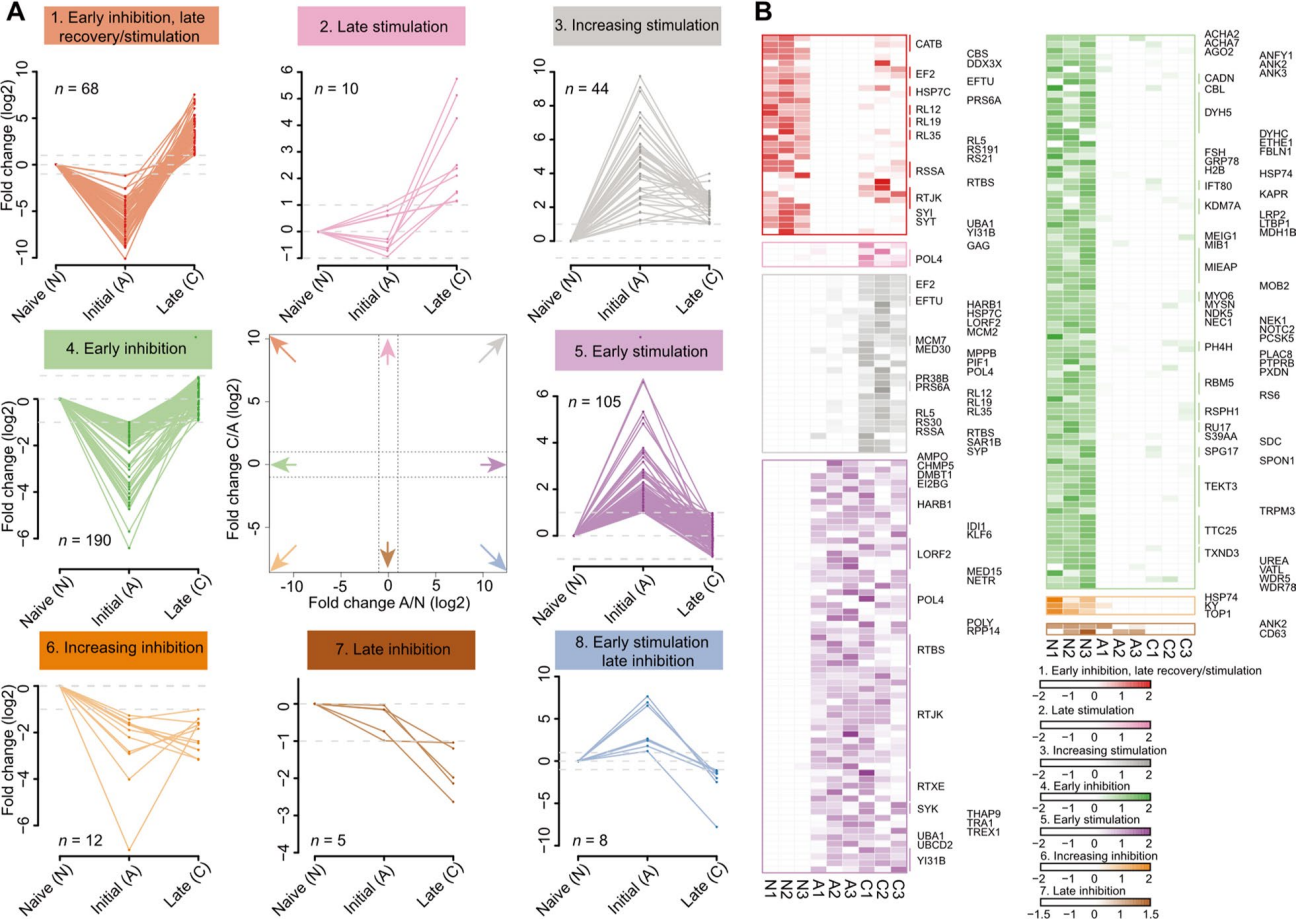
Dynamic gene expression of snails with schistosome infection. **A** Analysis of coincident gene expression dynamics using data from naive snails (*Oncomelania hupensis*) and snails in the initial and late stages of *S. japonicum* infection (PRJNA551328) and naive and *S. mekongi*-infected snails (*N. aperta*). *n* represents the number of unigenes belonging to the cluster; **B** Heatmap of differentially expressed genes enriched in GO terms. The gene symbols with colored lines represent different unigenes annotated to the same gene

To understand the effects of *S. mekongi* infection on *N. aperta*, we next performed a comparative RNA-seq analysis of snail tissues infected with or without *S. mekongi*. Through a systematic de novo assembly pipeline, a total of 4840 unigenes were observed to be differentially expressed (551 upregulated and 4289 downregulated, |GFOLD|> 2). To validate the observed expression pattern and reduce the limitation imposed by sample size, we next incorporated the critical genes involved in the *O. hupensis* response to *S. japonicum* infection based on the same pipeline due to the lack of a reference genome [82]. In total, 98 up-regulated and 133 down-regulated genes were observed in both *S. mekongi*- and *S. japonicum*-infected snails. Interestingly, a decreased expression level of TLR2-encoding genes was observed in our infected snails at a later stage. Our further clustering analysis distinguished all differentially expressed genes into eight expression patterns (Fig. 6A, different unigenes may be annotated to the same gene). Accordingly, GO enrichment analysis displayed [83] that several metabolic and biosynthetic processes were significantly enriched with up-regulated genes, while developmental and differentiation-related processes were enriched with down-regulated genes (Additional file 1: Fig. S9).

## Discussion

Extensive genomic studies have explored three major blood flukes (*S. japonicum, S. mansoni*, *and S. haematobium*), but studies related to *S. intercalatum* and *S. mekongi* are still limited [14, 17–19, 21]. Here, we employed multiple technologies, such as PacBio and HiC technologies, to generate a chromosome-level reference genome of *S. mekongi*.

We produced a high-quality chromosome-level genome for *S. mekongi*, and the phylogenetic analysis of 12 flatworms showed that *S. mekongi* is sister to *S. japonicum* and possesses the baseline characteristics of schistosome species. The obtained genome of *S. mekongi* provides a critical resource for investigating schistosomiasis transmission and comparative genomics. The chromosome-level synteny of *S. mekongi* and *S. mansoni* showed macrosynteny with local rearrangement, providing some clues for understanding their divergence. Our results suggested that the low conserved region might have originated in the ancestral genome of Schistosomatidae and undergone local rearrangement in other blood fluke genomes. Consequently, the comparative genomic data from this investigation should be used as a reference for future functional and large-scale population genomics and molecular studies of *S. mekongi*, with an emphasis on improved schistosomiasis public health interventions. Moreover, we are confident that our thorough analysis will contribute to elucidating historical events of introgression between or among closely related schistosome species and revealing proof of those occurrences.

*Schistosoma* species exhibit unique biological characteristics, including blood feeding, immune evasion, skin penetration and hermaphroditism. We found that several proteases and protease inhibitors were associated with these essential phenotypes of *S. mekongi* (Additional file 1: Table S11). Brady et al. demonstrated that the peptidase cathepsin-L (C01A) is dominantly responsible for the degradation of host hemoglobin by *S. japonicum* and *S. mansoni* [58]. Previous studies have illustrated that *S. japonicum* lysophospholipase, belonging to the S09X family, can disrupt the conformation of the host cell membrane when pathogens invade and to induce cell lysis [59, 60]. Lysosomal aspartic proteases belonging to subfamily A01A function under acidic conditions and show expanded substrate specificity [61]. Similarly, we found that peptidases in family M28 that participate in the synthesis of glucose and lipids could affect the process of antigen disguise and the presentation of information, probably leading to immune evasion by *S. mekongi* [14]. In addition, modulators in the I93 peptidase family encoding components of the Wnt pathway are associated with the growth and development of *Schistosoma* species [62]. Our explored diverse proteases (degradome) implicated in almost all physiologic and pathological processes may therefore hold promising therapeutic potential. Some studies involving strong drug discovery efforts targeting above mentioned peptidases are already in place to develop anti-schistosomal treatment [7, 14]. To identify potential differentiation-related genes, we scanned the homologs of sexual development-related genes from *S. japonicum* in *S. mekongi* (Additional file 1: Table S12). GO enrichment showed that a mass of the genes was involved in vitelline development processes, associated with positive regulation of cell proliferation and lysosomal membrane. However, for a thorough investigation of sex-determining and developmentally-regulated gene functions (expression), additional study is required to collect long-read data from male and female *S. mekongi*.

A comparative analysis of 12 flatworms, including one free-living species, three parasitic tapeworms, three liver flukes and five blood flukes, revealed specific molecules and expanded proteases, such as CEs and leishmanolysin-like metalloprotease M8. CEs are serine proteases deposited in advance of parasite invasion by the holocytosis of vesicles from ten large acetabular gland cells [63]. One CE copy has been reported in *S. japonicum*, while multiple copies were found in other schistosome species, such as *S. mansoni* and *S. haematobium* [14, 63]. Intriguingly, in the present study, we detected two recently duplicated copies of CE and multiple divergent CEs in other blood flukes, particularly in *S. mansoni*. Exploring these diverse elastases will contribute to better understanding the biology of *S. mekongi* and identifying gene targets to make it easier to find potential drug and vaccine candidates. For illustration, in a recent study, cost-effective biosensors for the early and rapid detection of schistosome parasites were based on elastases released by the *S. mansoni* cercarial larval stages [64]. A leishmanolysin-like metalloprotease (M8), also known as Gp63 or SmLeish, is the excretory/secretory product of *S. mansoni* and is produced by sporocysts during snail infection, which can aid sporocyst encapsulation to promote survival in intermediate hosts. In a recent study, prior to exposure to snails (*Biomphalaria glabrata*), SmLeish knockdown in *S. mansoni* miracidia significantly lowered miracidia penetration success, delaying the onset of infections in the patient and reducing the production of cercaria from snails (infected) [72]. M8 is also critical for *S. japonicum* cercariae to penetrate the host's skin [82]. The expansion of these enzymes in *S. mansoni* has also been reported, but the evolution of M8 in flatworms remains limited [83]. Independent expansion of M8 was observed in liver flukes and blood flukes. Lineage-specific M8 was presented in blood flukes. The inference of M8 expression in cercariae and schistosomulae from the *T. regenti* transcriptome showed specifically high expression in cercariae. Combined with the results from the functional investigation of M8 in *S. mansoni* [65], we hypothesized that the presence of M8 in flukes is related to their survival in intermediate hosts. In other words, M8 may serve as an ideal candidate for drug and vaccine development so that the growth cycle of cercariae may be interrupted and consequently to break the disease transmission. However, the reason why extensively duplicated and divergent M8 enzyme is retained in blood flukes remains unclear and requires additional functional experiments (e.g., RNAi experiments).

Blood flukes require snails as an intermediate host to produce hundreds of infective cercaria that penetrate the skin of definitive hosts, including humans, causing schistosomiasis. The phylogenetics and habitat diversity of the snail intermediate host suggested that *S. mekongi* might be more widespread and that the risk to public health is up to ten times higher than had been presumed previously [8]. Snail control is a priority for reducing schistosomiasis transmission [66]. Additionally, host-parasite interactions may impact the parasite's genome [84, 85]. Understanding the interaction between the intermediate host is of significant importance for the prevention schistosomiasis. Notably, a previous study mentioned that the expression of Toll-like receptors (TLRs) in the intermediate host of *S. japonicum*, *O. hupensis*, was up-regulated in the initial stage but inhibited later during *S. japonicum* infection [86]. Moreover, similar pathways, such as the pyrimidine metabolism pathway and cholesterol biosynthesis, have been shown to be enriched based on differentially expressed genes in *O. hupensis* after *S. japonicum* infection [82]. These results suggested that schistosome infection might facilitate metabolic and biosynthetic activity and reduce the growth ability of intermediate hosts to support the production of cercaria. *N. aperta* is recognized as the only intermediate host of *S. mekongi*, which plays a vital role in the life cycle of this parasite [87]. Similarly, a decreased expression level of TLR2-encoding genes of *N. aperta* was decreased at later infection stage, and up-regulated genes were significantly enriched in some metabolic and biosynthetic processes. Taken together, these findings made a substantial contribution to our knowledge of a number of genes involved in the innate immunological responses of different host snails and parasite-host cross-talk, subsequently leading to the development of new markers for the identification of infected snails. It may offer new perspectives on *Schistosoma* public health intervention by understanding the kinetics and dynamics of snail immune-parasite infection establishment.

Our findings will offer insights for use in future studies on the origins, radiation, phylogenetic association among the populations and other related taxa, and potential dispersal abilities of *S. mekongi*. Also, it may further confirm the species involved at each known Mekong schistosomiasis transmission focus.

Admittedly, one limitation of the current study is the small sample size, which restricted more extensive investigations, including gene function analysis and RNA sequencing of *S. mekongi* at various developmental stages in snails. To address this, future research should focus on examining the roles and mechanisms of unique genes and observing changes in gene expression of the parasite during different stages of development within its hosts.

## Conclusions

In this study, we present the first high-quality, chromosome-scale genome assembly of the human blood fluke, *S. mekongi*. This breakthrough paves the way for comprehensive genetic analysis of various schistosomes, addressing key questions about their genetics, evolution, ecology, pathophysiology, epidemiology, and host-parasite interactions. Insights from our genomic data have significant potential for advancing the development of vaccines, anti-schistosome drugs, and diagnostic methods. Further, our results may help future studies investigating gene function and essentiality employing techniques, i.e., RNA interference and transgenesis.

### Supplementary Information

**Additional file 1.** Supplementary figures and tables.

## Data Availability

The datasets presented in this study can be found in online repositories. The names of the repository/repositories and accession number(s) can be found below: https://www.ncbi.nlm.nih.gov/genbank/, the assembled genome data and the RNA-seq data have been submitted to NCBI (Bioproject accession number PRJNA803609). Other data used to support the findings of this study are available from the corresponding author upon request.

## Abbreviations

Hi-C: High-throughput chromosome conformation capture
BUSCO: Benchmarking Universal Single-Copy Orthologs
LTRs: Long terminal repeats
UTRs: Untranslated regions
TSDs: Target site duplications
FPKM: Fragments per kilobases per million reads
SRA: Sequence read archive
NCBI: National Center for Biotechnology Information
LINEs: Long interspersed nuclear elements;
mt: Mitochondrial
TEs: Transposable elements
GC: Guanine-cytosine
CEs: Cercarial elastases
GO: Gene ontology
KEGG: Kyoto Encyclopedia of Genes and Genomes
TLRs: Toll-like receptors
