# Supplementary material for: Chromosome-scale genome of the human blood fluke Schistosoma mekongi and its implications for public health
Source: Infect Dis Poverty. 2023 Nov 28;12:104. doi: 10.1186/s40249-023-01160-6 (PMC10683246; doi:10.1186/s40249-023-01160-6)
Supplement: Supplementary file 1 — Additional file 1. Supplementary figures and tables. [file 40249_2023_1160_MOESM1_ESM.zip › Additional file 1/Figure S1.docx]

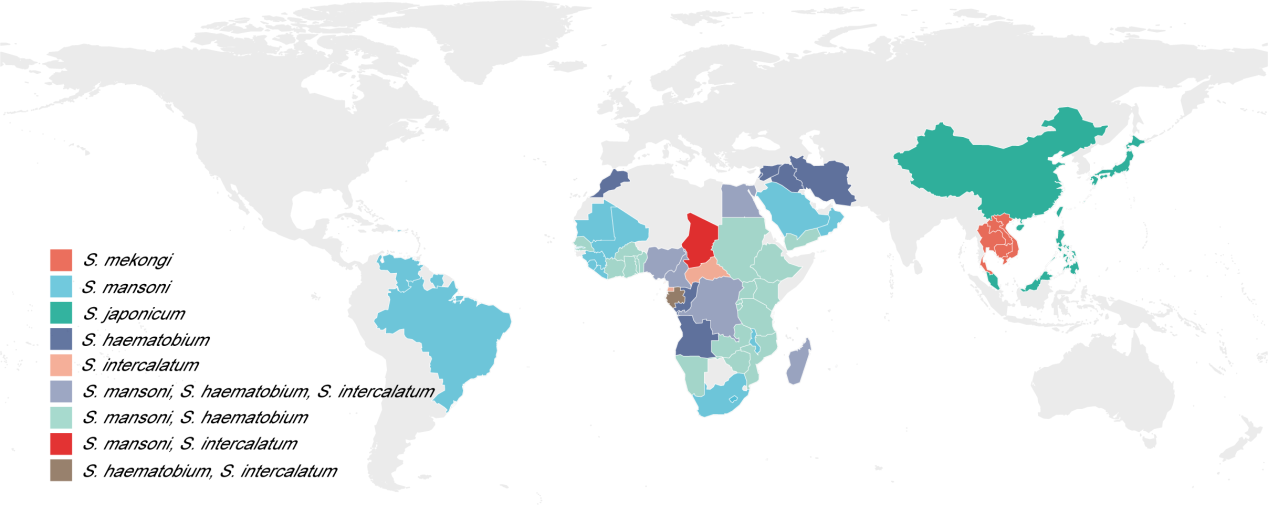


**Figure S1.** **Distribution of human blood flukes**

[*S*](https://www.sciencedirect.com/topics/immunology-and-microbiology/schistosoma)*. mekongi* is distributed in Thailand, Vietnam, Laos, and Cambodia in the Mekong River Basin. [*S*](https://www.sciencedirect.com/topics/immunology-and-microbiology/schistosoma)*. japonicum* is distributed in China, Japan, Malaysia, and Philippines. [*S*](https://www.sciencedirect.com/topics/immunology-and-microbiology/schistosoma)*. mekongi* and [*S*](https://www.sciencedirect.com/topics/immunology-and-microbiology/schistosoma)*. japonicum* are not distributed in the same geographical area. *S. mansoni*, *S. haematobium*, and *S. intercalatum* are distributed in a large area of Africa. The data for this image were collected from published literature.
