# Supplementary material for: Chromosome-scale genome of the human blood fluke Schistosoma mekongi and its implications for public health
Source: Infect Dis Poverty. 2023 Nov 28;12:104. doi: 10.1186/s40249-023-01160-6 (PMC10683246; doi:10.1186/s40249-023-01160-6)
Supplement: Supplementary file 1 — Additional file 1. Supplementary figures and tables. [file 40249_2023_1160_MOESM1_ESM.zip › Additional file 1/Figure S8.docx]

**
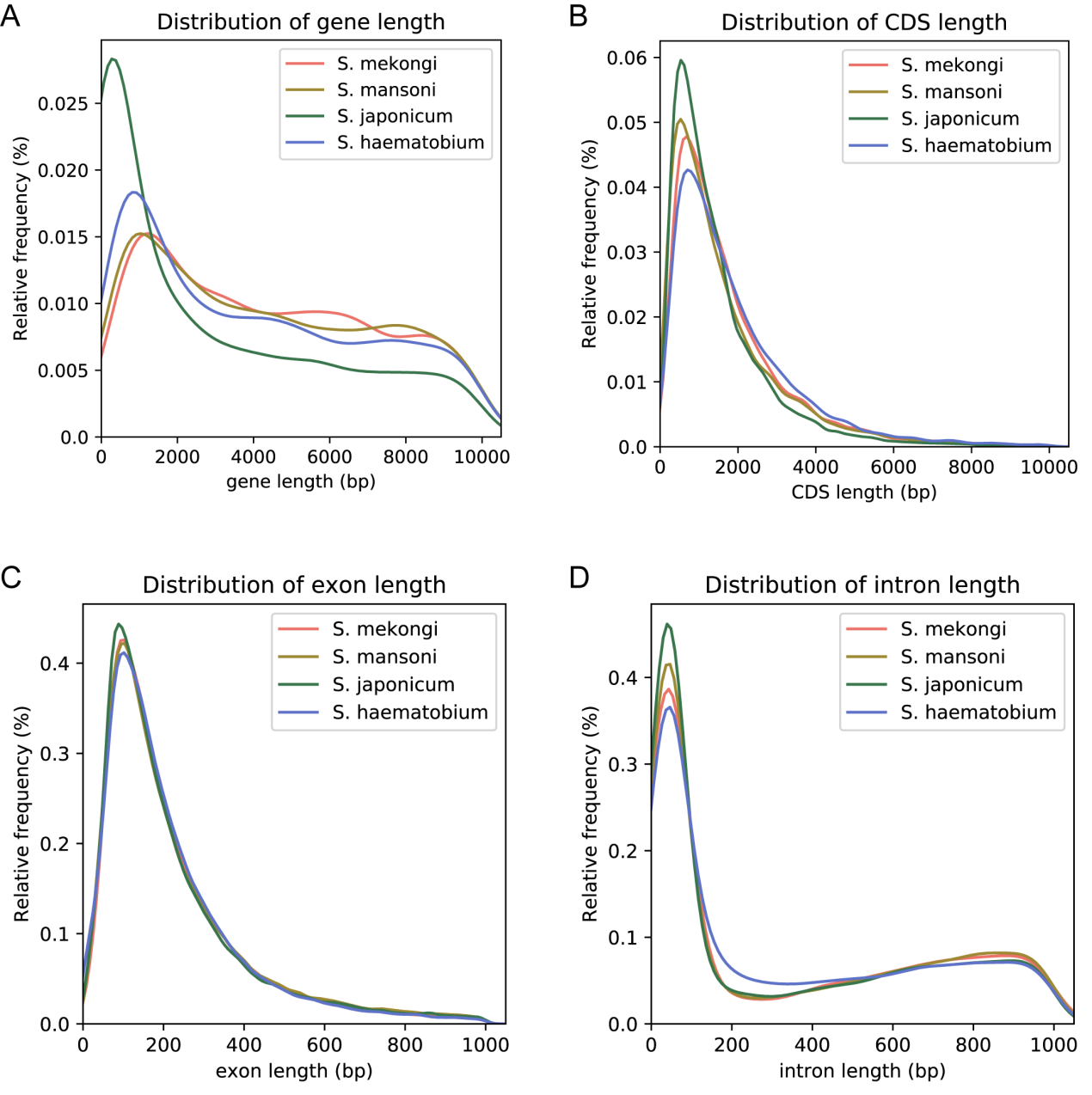
**

**Fig S8. Length distribution comparison on total gene, CDS, exon, and intron of annotated gene models of the *S. mekongi* and other human blood flukes.**

Length distribution of total genes (A), CDS (B), exon (C), and intron (D) were compared to those of *S. mekongi, S. mansoni, S. japonicum, and S. haematobium*.
