# Supplementary material for: Chromosome-scale genome of the human blood fluke Schistosoma mekongi and its implications for public health
Source: Infect Dis Poverty. 2023 Nov 28;12:104. doi: 10.1186/s40249-023-01160-6 (PMC10683246; doi:10.1186/s40249-023-01160-6)
Supplement: Supplementary file 1 — Additional file 1. Supplementary figures and tables. [file 40249_2023_1160_MOESM1_ESM.zip › Additional file 1/Figure S9.docx]

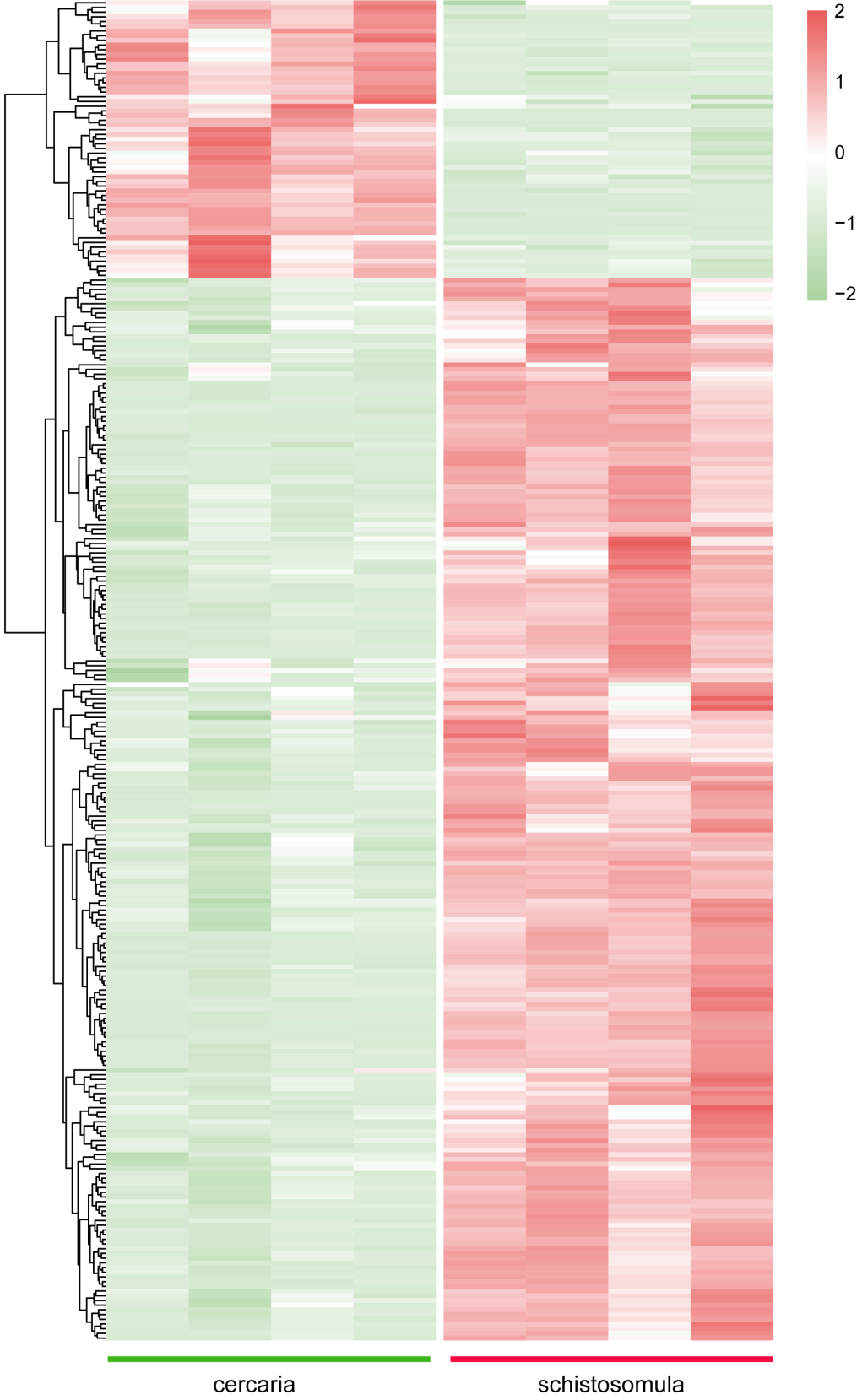


**Figure S9. Differential expression of unconserved genes between cercaria and schistosomula of *T. regent* (65 genes upregulated in cercaria and 220 genes upregulated in schistosomula) with one-to-one orthologue in *S. mekongi* based on reciprocal blast identification.**
