# Supplementary figures and images for: Chromosome-scale genome of the human blood fluke Schistosoma mekongi and its implications for public health
Source: Infect Dis Poverty. 2023 Nov 28;12:104. doi: 10.1186/s40249-023-01160-6 (PMC10683246; doi:10.1186/s40249-023-01160-6)

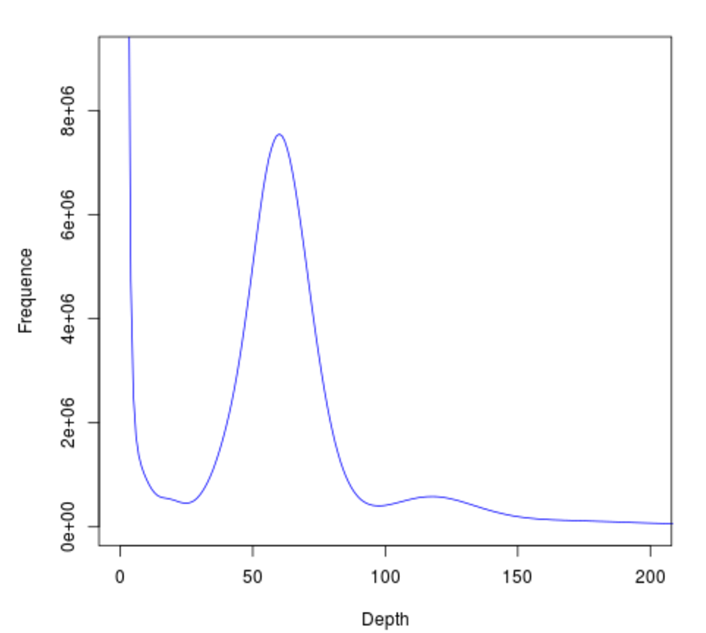


**Figure S2. 17-mer frequency of *S. mekongi* based on Illumina clean reads.**

Supplement: Supplementary file 1 — Additional file 1. Supplementary figures and tables. [file 40249_2023_1160_MOESM1_ESM.zip › Additional file 1/Figure S2.docx]

**
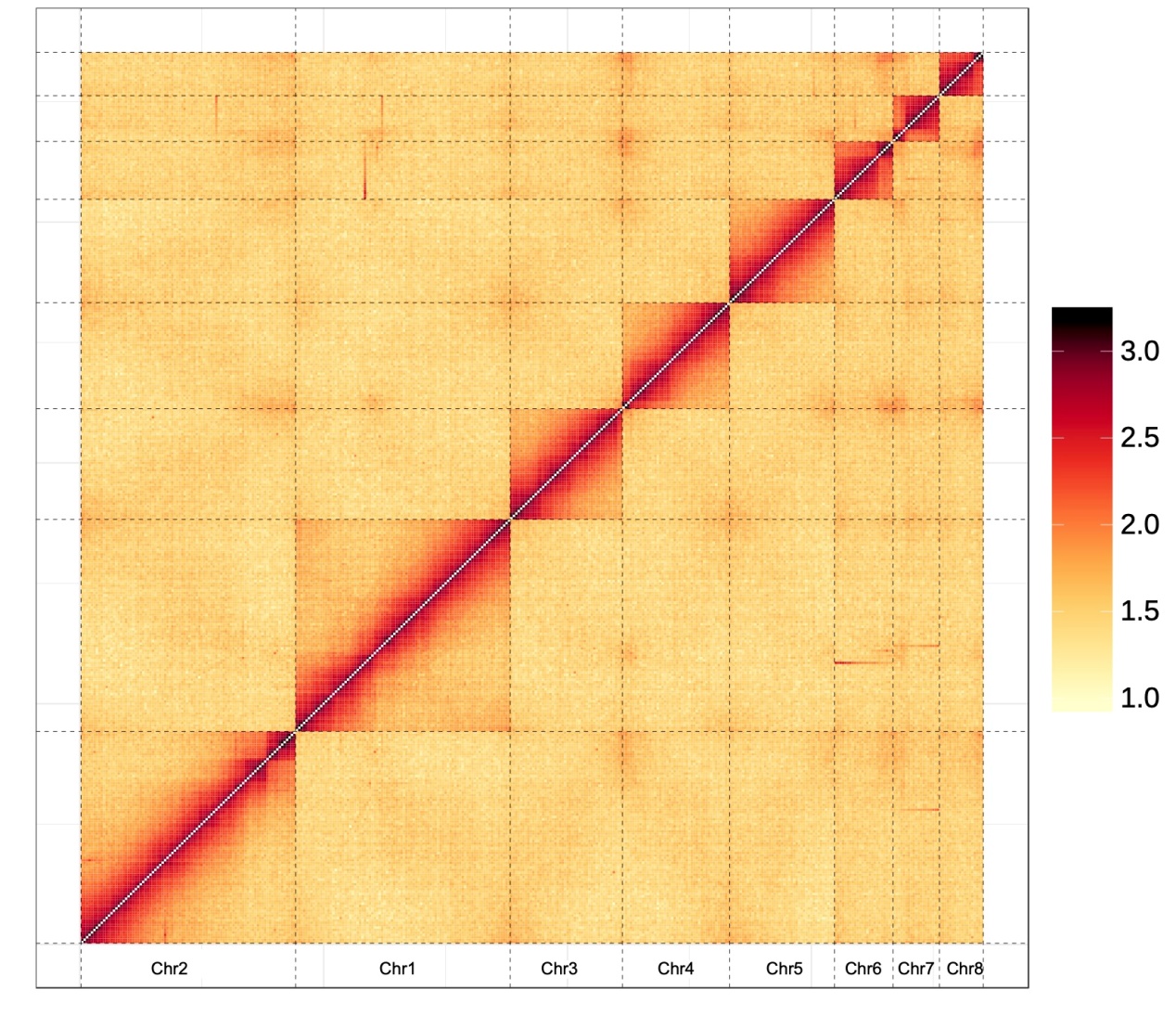
**

**Figure S3. Hi-C produces a genome-wide contact matrix between eight pseudochromosomes.**

Supplement: Supplementary file 1 — Additional file 1. Supplementary figures and tables. [file 40249_2023_1160_MOESM1_ESM.zip › Additional file 1/Figure S3.docx]

**
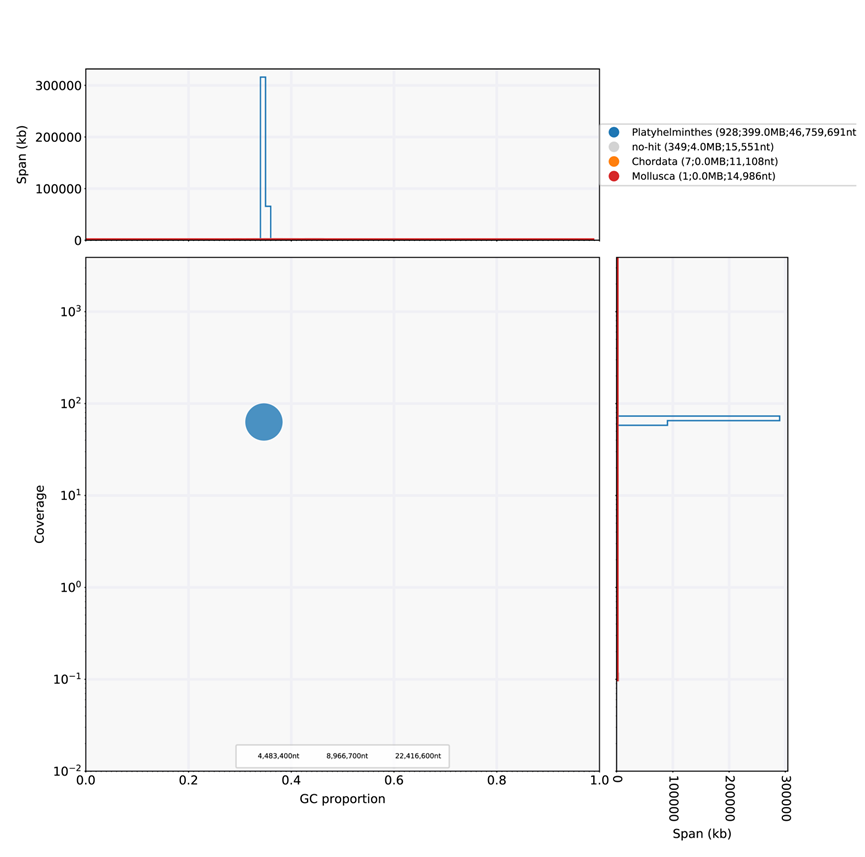
**

**Figure S4. Containment sequences evaluation using BlobTools software.**

Supplement: Supplementary file 1 — Additional file 1. Supplementary figures and tables. [file 40249_2023_1160_MOESM1_ESM.zip › Additional file 1/Figure S4.docx]

**
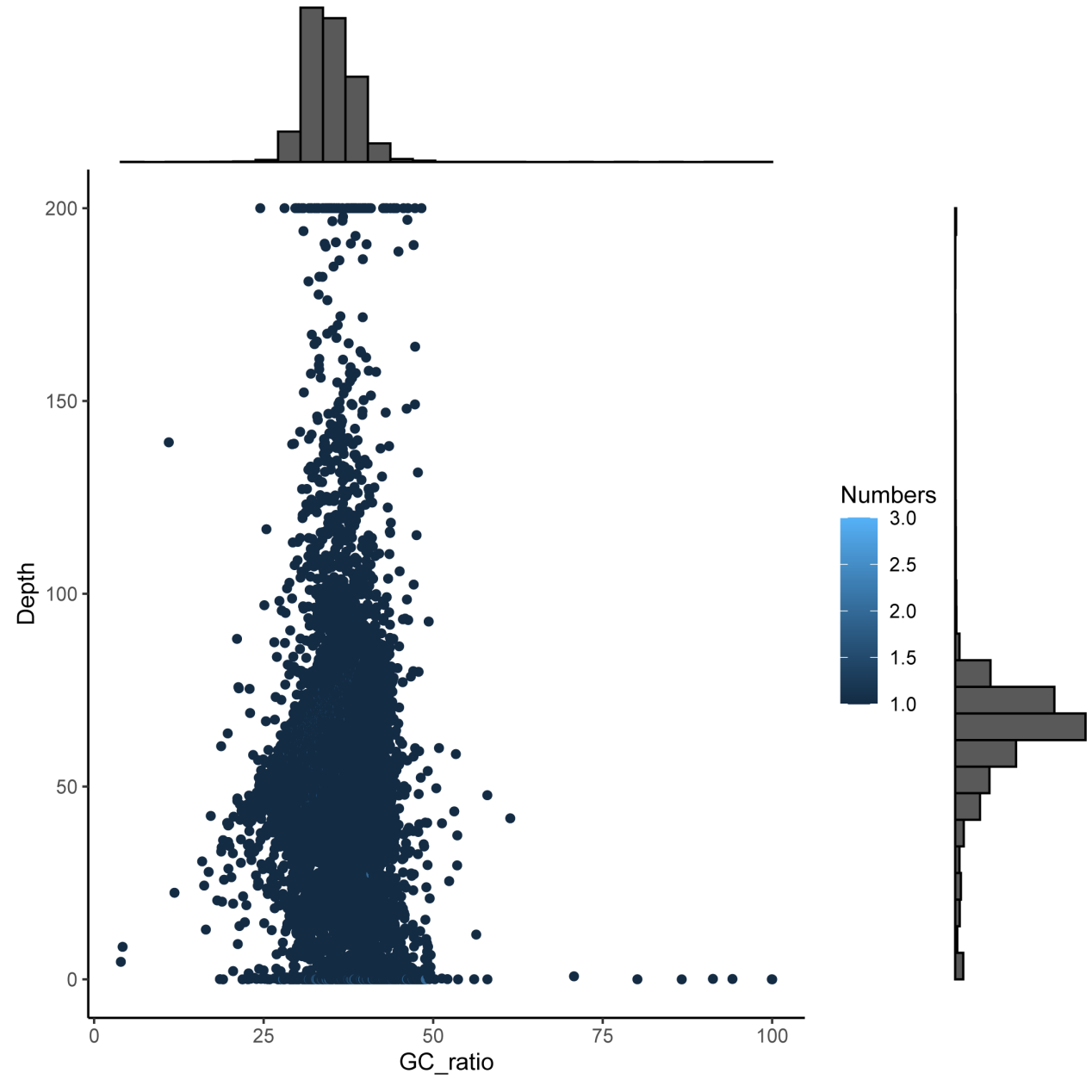
**

**Figure S5. The distribution of GC content and depth.**

Supplement: Supplementary file 1 — Additional file 1. Supplementary figures and tables. [file 40249_2023_1160_MOESM1_ESM.zip › Additional file 1/Figure S5.docx]

**
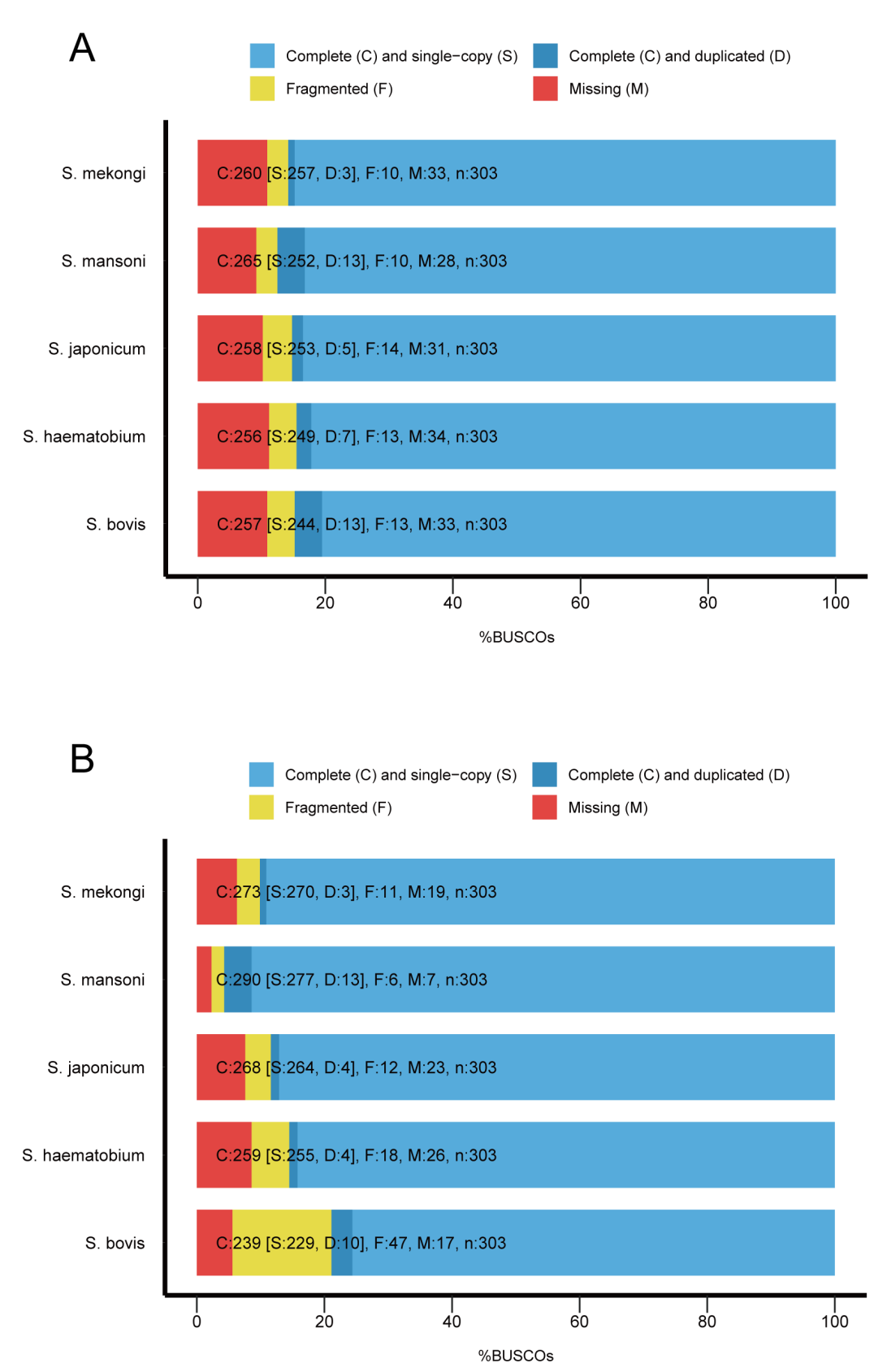
**

**Figure S6. (A) Genome-level and (B) Protein-level completeness estimation using BUSCO pipeline**

Supplement: Supplementary file 1 — Additional file 1. Supplementary figures and tables. [file 40249_2023_1160_MOESM1_ESM.zip › Additional file 1/Figure S6.docx]

**
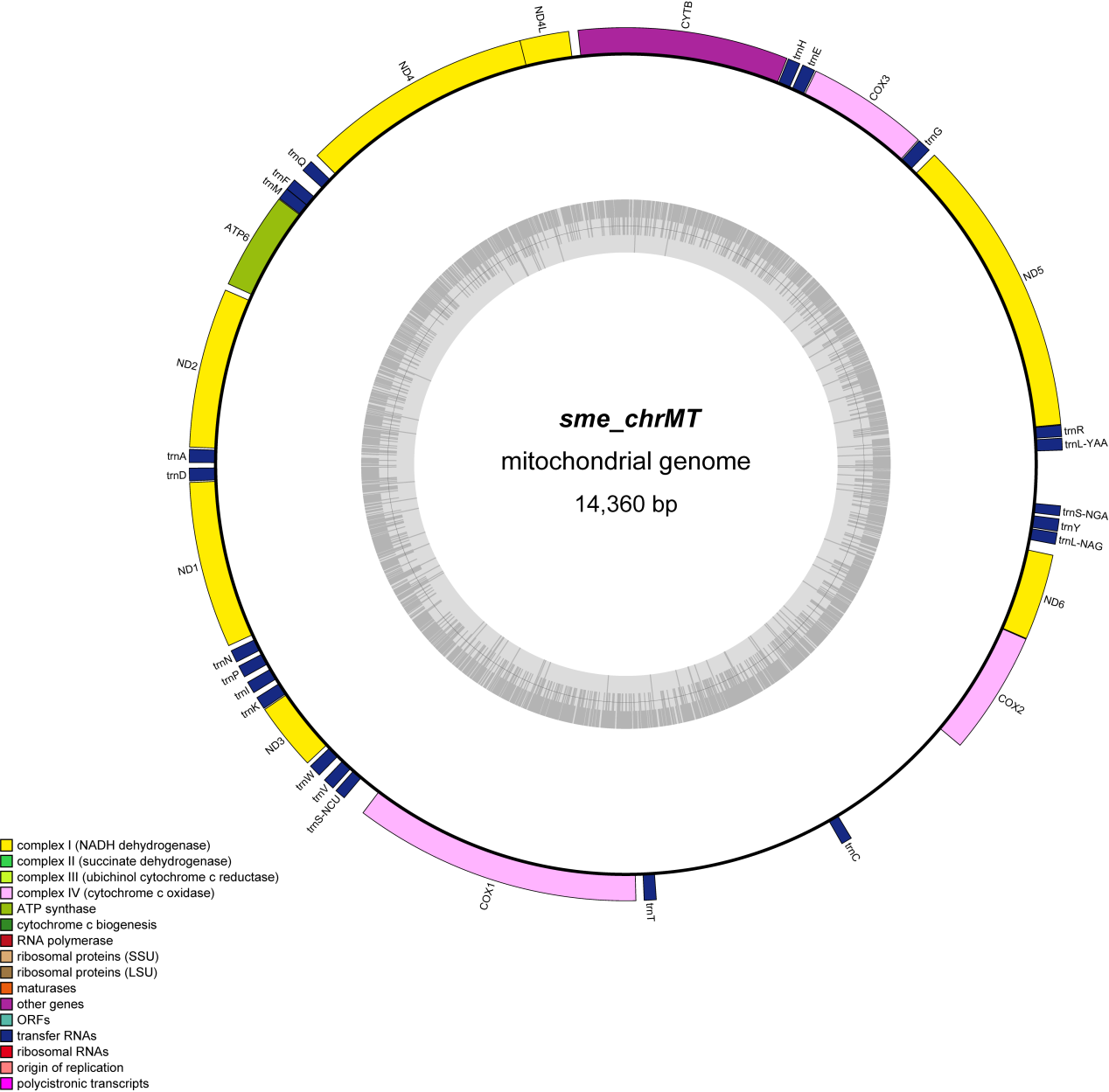
**

**Figure S7. The assembled mitochondria genome using PacBio long reads.**

Supplement: Supplementary file 1 — Additional file 1. Supplementary figures and tables. [file 40249_2023_1160_MOESM1_ESM.zip › Additional file 1/Figure S7.docx]
